# IgE binds asymmetrically to its B cell receptor CD23

**DOI:** 10.1038/srep45533

**Published:** 2017-03-31

**Authors:** Balvinder Dhaliwal, Marie O. Y. Pang, Anthony H. Keeble, Louisa K. James, Hannah J. Gould, James M. McDonnell, Brian J. Sutton, Andrew J. Beavil

**Affiliations:** 1 Division of Infection, Immunity and Respiratory Medicine, School of Biological Sciences, Faculty of Biology, Medicine and Health, Manchester Academic Health Sciences Centre and Manchester Institute of Biotechnology, University of Manchester, 131 Princess Street, Manchester, M1 7DN, U.K.; 2 Randall Division of Cell and Molecular Biophysics, Faculty of Life Sciences & Medicine, King’s College London, New Hunt’s House, Guy’s Campus, London, SE1 1UL, U.K.; 3 Medical Research Council and Asthma UK Centre in Allergic Mechanisms of Asthma, London, U.K.

## Abstract

The antibody IgE plays a central role in allergic disease mechanisms. Its effector functions are controlled through interactions between the Fc region and two principal cell surface receptors FcεRI and CD23. The interaction with FcεRI is primarily responsible for allergic sensitization and the inflammatory response, while IgE binding to CD23 is involved in the regulation of IgE synthesis and allergen transcytosis. Here we present the crystal structure of a CD23/IgE-Fc complex and conduct isothermal titration calorimetric binding studies. Two lectin-like “head” domains of CD23 bind to IgE-Fc with affinities that differ by more than an order of magnitude, but the crystal structure reveals only one head bound to one of the two identical heavy-chains in the asymmetrically bent IgE-Fc. These results highlight the subtle interplay between receptor binding sites in IgE-Fc and their affinities, the understanding of which may be exploited for therapeutic intervention in allergic disease.

An estimated 40% of the population suffer from at least one form of allergy, with their prevalence continuing to soar worldwide^1^. Allergic diseases, which include allergic rhinitis (‘hay fever’), respiratory allergy (*e.g.* atopic asthma) and food allergy, have a detrimental impact on a person’s quality of life^2^,^3^,^4^,^5^, a nation’s economy^6^,^7^, and in acute cases can be life threatening^8^.

The antibody immunoglobulin E (IgE) plays a central role in allergic disease mechanisms. For reasons poorly understood, IgE recognizes seemingly innocuous substances known as allergens *via* its Fab regions, whilst its effector functions are controlled through interactions of the Fc region with two principal cell surface receptors, FcεRI and CD23 (also known as FcεRII)^9^,^10^. FcεRI, found on mast cells and basophils, binds IgE with high affinity (*K*_D_ of 0.01 to 0.1 nM)^11^ and is responsible for allergic sensitization and the immediate hypersensitivity response in which minute amounts of allergen crosslink FcεRI-bound IgE triggering cellular degranulation, release of proinflammatory mediators such as histamine and the initiation of an allergic response^12^.

The second receptor for IgE, CD23, is unique among Ig receptors in that it belongs to the C-type lectin-like superfamily^13^. Expressed in several hematopoietic cell types, including B cells and antigen-presenting cells, in its membrane-bound form CD23 consists of three C-type lectin ‘head’ domains connected to the membrane by a trimeric α-helical coiled-coil ‘stalk’^14^. A single head domain binds to human IgE-Fc with an affinity *K*_D_ of ~1 μM^15^, although avidity of the trimer^16^,^17^,^18^,^19^ and the presence of Ca^2+^ 15,20 can substantially enhance the interaction. The stalk region of CD23 is susceptible to attack by endogenous proteases such as ADAM10^21^, releasing soluble trimeric and monomeric forms of CD23. The house dust mite allergenic protease *Der p* 1 generates a soluble monomeric form of CD23 consisting of just the lectin head domain, termed derCD23^22^.

The interaction of CD23 with IgE is involved in regulating many allergic processes^23^. For example, when expressed on epithelial cells, CD23 is involved in the transportation of IgE-allergen immune complexes across the gut^24^ and airways^25^. On B cells that have switched to IgE production, the CD23-IgE interaction is critical to both the up- and down-regulation of IgE synthesis depending on the oligomeric form of CD23^9^. This interaction is also essential in the process of facilitated antigen presentation (FAP)^9^, in which IgE-allergen complexes are internalized by CD23 on antigen-presenting cells. The allergen is then proteolytically cleaved and its peptides complexed with MHC class II molecules and recycled to the cell surface for Type 2 helper (T_H_2) cell recognition and activation. The activated T_H_2 cells secrete cytokines that ultimately lead to an increase in IgE synthesis and exacerbation of allergic inflammation^26^,^27^. Recent evidence indicates that the CD23 surface density on B cells determines its FAP activity^28^. The CD23-IgE interaction is thus involved in both the development and regulation of the allergic response, and indeed, molecules designed to target the interaction such as the anti-CD23 IgG antibody Lumiliximab and DARPins have been shown to inhibit IgE synthesis^29^,^30^. A molecular understanding of the CD23-IgE interaction thus has implications for the control of allergic disease.

We previously reported the crystal structure of derCD23 in complex with Fcε3-4^31^, a subfragment of IgE-Fc consisting of a dimer of the Cε3 and Cε4 domains. One head domain was found to bind to each heavy chain of IgE, principally to the Cε3 domains but with a contribution from Cε4, explaining the known 2:1 stoichiometry of monomeric CD23 binding to both IgE-Fc and Fcε3-4^19^,^32^. Although the binding sites for FcεRI and CD23 lie at opposite ends of the Cε3 domain, we went on to demonstrate that the binding of the two receptors to IgE are allosterically linked and mutually exclusive, with conformational changes in Fcε3-4 upon CD23 binding precluding the binding of FcεRI and *vice versa*^31^.

Here we present the crystal structure of derCD23 in complex with the complete IgE-Fc region, including the Cε2 domains, which in the free molecule fold back onto the Fcε3-4 region in an asymmetrical fashion^33^. One derCD23 molecule is seen binding to one IgE-Fc chain, making contact with all three domains. Isothermal titration calorimetry (ITC) experiments confirm the 2:1 (derCD23:IgE-Fc) stoichiometry in solution and reveal that the two binding affinities differ by more than an order of magnitude; this is explained in terms of the crystal structure.

## Results

### Quaternary structure of the derCD23/IgE-Fc complex

**Figure 1:**
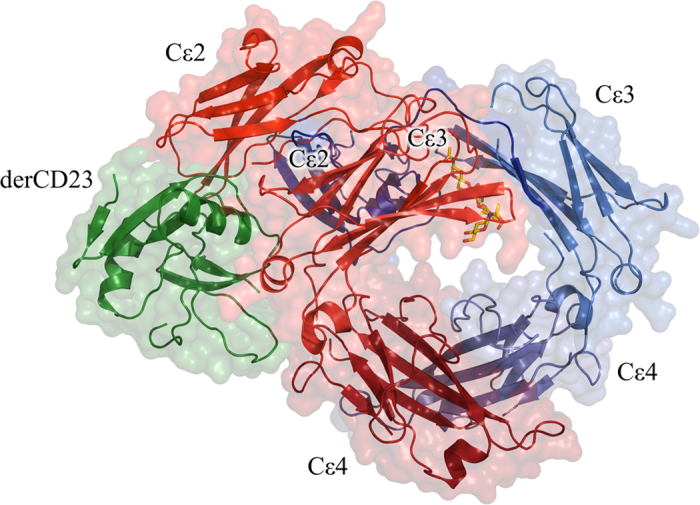
Structure of the 1:1 derCD23/IgE-Fc complex. The derCD23 (green Cα traces with surfaces) binds to one heavy chain of IgE-Fc (red and blue) contacting the Cε3 and Cε4 domains. The Cε2 domains are asymmetrically bent back onto one Cε3 domain and make some additional contacts with derCD23. The carbohydrate is shown in all-atom representation (red and yellow, without surfaces) and can be seen behind the Cε3 domain.

The crystal structure contains two essentially identical complexes of derCD23/IgE-Fc within the asymmetric unit. Each complex consists of one IgE-Fc molecule bound to one molecule of derCD23 (Fig. 1). The derCD23 molecule interacts with the IgE-Fc chain onto which the Cε2 domains are folded back. The 1:1 stoichiometry of the complex (*i.e.*, one IgE-Fc dimer bound to one molecule of derCD23) was unexpected, as it is well documented that one CD23 head domain binds to each heavy chain of IgE, whether the Fcε3-4 sub-fragment or the complete IgE-Fc^15^,^19^,^31^,^32^.

### The derCD23/IgE-Fc interface

**Figure 2:**
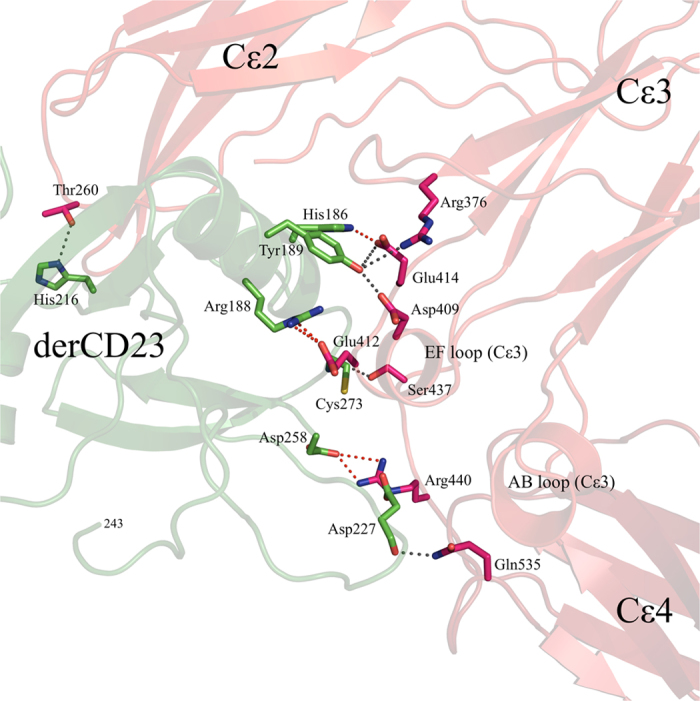
Salt bridges and hydrogen bonds at the derCD23/IgE-Fc interface. The five H-bonds associated with salt bridges in both independent complexes are shown in red, and the six remaining H-bonds are shown in black.

The derCD23 molecule binds principally to the Cε3 domain with lesser interactions with Cε4 and the short Cε3-Cε4 linker region. The interface is predominantly hydrophilic, consisting of five salt bridges with hydrogen bonds, and six additional hydrogen bonds (Fig. 2). The majority of these interactions are similar to those observed at the interface of the derCD23/Fcε3-4 crystal structure^31^, as well as being consistent with NMR chemical shift perturbation studies and binding experiments using proteins with mutated interface residues^34^. There is an additional interaction between Asp258 of CD23 and Arg440 in the Cε3-Cε4 linker, an interaction that had only previously been observed in the Ca^2+^-bound derCD23/Fcε3-4 crystal structure (Figure S1). In the absence of Ca^2+^ binding to CD23, as in the complex presented here, the loop including Asp258 is found to be partially flexible^20^,^31^,^35^,^36^. Despite the close approach of derCD23 to the Cε2 domain in the same heavy chain, just a single hydrogen bond is observed between His216 of derCD23 and Thr260 of Cε2 (Figs 1 and 2).

The derCD23/IgE-Fc interface shows an even poorer surface complementarity^37^ than that found in the derCD23/Fcε3-4 complex; the calculated S_c_ values^38^ range between 0.49–0.51 for the former, compared to 0.63–0.71 for the latter (the range of values refers to the different molecular complexes in the asymmetric unit). The buried surface area of the derCD23/IgE-Fc interface ranges from 965 to 1042 Å^2^, with relative contributions from the Cε2 domain of 24%, the Cε3 domain 56%, the Cε3-Cε4 linker (Ser437 to Arg440) 10%, and the Cε4 domain 10%. The Cε2-Cε3 linker (Lys326 to Ser337) does not contribute to the interface. Relatively few well-defined residues are involved in the network of van der Waals contacts at the interface (Gly180, Trp184, Val185, Phe272 and Asp274 in derCD23; Phe321, Ser378, Lys380, Ile411 and Lys435 in IgE-Fc). Of these hydrophobic interactions, only one involves the Cε2 domain: Phe321 interacts with Gly180 of derCD23. No interactions are observed between derCD23 and the other IgE-Fc heavy chain.

### Comparison with previous IgE-Fc structures

**Figure 3:**
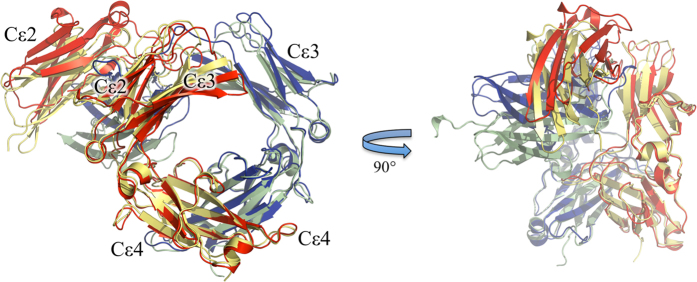
Orthogonal views depicting conformational changes in IgE-Fc upon binding a single molecule of derCD23 (not shown). A substantial rigid body movement of the IgE-Fc Cε2 domains upon derCD23 binding is observed when compared to free IgE-Fc (yellow and green). In addition, the Cε3 domain in the derCD23-bound chain (red) adopts an extreme closed conformation, in contrast to the Cε3 domain in the free chain (blue), which adopts an extreme open conformation. The structures were superposed on their (Cε4)_2_ domains.

IgE-Fc strongly favors an asymmetrically bent structure in its free state^39^, with the Cε2 domain pair folded back and making contact not only with one of the Cε3 domains but also Cε4^33^. Previous modeling of the derCD23/IgE-Fc complex by docking the (Cε2)_2_ domain pair from the free IgE-Fc structure onto the derCD23/Fcε3-4 complex had suggested that only minor rearrangements of a few N-terminal residues in the Cε2 domain were required to prevent a steric clash with derCD23^31^. However, the experimentally determined complex shows that upon binding of a single derCD23 molecule, the disulfide-linked (Cε2)_2_ domain pair move as a rigid unit, swinging out 16° to accommodate the derCD23 molecule (Fig. 3 and Supplementary movie).

The Fcε3-4 moiety of the IgE-Fc dimer in the complex also adopts a surprising conformation upon derCD23 binding. Flexibility in the Cε3-Cε4 interdomain angle has been well documented in both unliganded Fcε3-4^40^,^41^ and IgE-Fc^33^, and in complex with sFcεRIα^11^,^42^ or derCD23^20^,^31^,^36^. A comparison of all previous Fcε3-4 and IgE-Fc structures shows that the Cε3-Cε4 angle varies over a range of 25°, with IgE-Fc or Fcε3-4 in complex with sFcεRIα adopting the most ‘open’ conformation for the Cε3 domains, and Fcε3-4 in complex with derCD23 adopting the most ‘closed’ conformation. Examination of the Cε3-Cε4 interdomain angles of IgE-Fc in complex with derCD23 presented here reveals that the chain bound to derCD23 adopts a closed conformation. In contrast, the free IgE-Fc chain unexpectedly adopts a very open conformation; indeed, the Cε3-Cε4 interdomain angles of the two chains differ by 16° (Figure S2). Comparison of the two IgE-Fc chains in the complex with all other IgE-Fc and Fcε3-4 structures shows that the derCD23-bound IgE-Fc chain has the same closed Cε3-Cε4 interdomain angle previously observed in the Ca^2+^-free derCD23-Fcε3-4 complex crystallized in space group *P*1^36^ (Figure S2). This closed conformation is clearly incompatible with FcεRI binding^31^,^42^. The free IgE-Fc chain, on the other hand, adopts the most open conformation observed, the same conformation previously only seen in complex with sFcεRIα^11^,^42^. Earlier structural studies had indicated that free IgE-Fc chains favoured an intermediate position between the open and closed conformations^33^, however our results reveal that the free chain of IgE-Fc adopts an extremely open conformation when the other chain is bound to derCD23: clearly a conformational change involving a closing of the Cε3-Cε4 interdomain angle is required to bind a second derCD23 molecule (Figure S3).

### The two CD23 binding sites on IgE have different affinities

The binding affinity of derCD23 to IgE-Fc was determined using ITC, by titrating derCD23 into a fixed concentration of IgE-Fc. The data fitted best to a two-site binding model (Figure S4). One binding site has an affinity of 1.2 μM (*K*_D1_; Table 1), while the other is over an order of magnitude (12-fold) weaker with an affinity of 14.4 μM (*K*_D2_).

## Discussion

The interaction of CD23 and IgE is critical in many allergic disease processes, including the regulation of IgE synthesis, facilitated allergen presentation and allergen transportation across the gut and airways. Here we present the crystal structure of a lectin-like head domain of CD23 in complex with the complete Fc receptor-binding region of IgE. The results of ITC experiments reported here confirm the 2:1 stoichiometry reported earlier^19^,^32^, but now reveal that the affinities for the two sites, one in each heavy chain, are very different (*K*_D_ = 1.2 μM and 14.4 μM), with distinct thermodynamic profiles (Table 1). This is consistent with results from an earlier sedimentation equilibrium study of derCD23 binding to Fcε3-4, which concluded that the two sites were thermodynamically distinct, with a greater enthalpy change for one site than the other, although the affinities were similar (*K*_D_ ~ 10 μM)^36^. The work reported here investigates the effect upon CD23 binding of the Cε2 domains in the complete IgE-Fc, which is known to have an acutely and asymmetrically bent structure in the free state^33^.

The crystal structure of derCD23 bound to IgE-Fc unexpectedly revealed only a 1:1 complex, with the single derCD23 molecule bound principally to the heavy chain onto which the Cε2 domains are folded back. The derCD23 molecule primarily contacts Cε3 and, as in the complexes with Fcε3-4^20^,^31^,^36^, also contacts Cε4 and the Cε3-Cε4 linker region; in addition however, there are a few contacts with Cε2 including a single hydrogen bond. The binding of this derCD23 molecule is also accompanied by a swinging out of the Cε2 domain pair (an “unbending” of the IgE-Fc) by 16°. Another aspect of the asymmetry of the IgE-Fc is the difference in the Cε3-Cε4 interdomain angle in the 1:1 complex: in the chain to which derCD23 is bound the angle is closed (as seen in the complexes with Fcε3-4), whereas in the other, free chain, the angle is very open and incompatible with derCD23 binding. This is in contrast to the earlier Fcε3-4 complexes in which two derCD23 molecules were bound symmetrically to the two chains, both of which adopted a closed interdomain conformation. Clearly further conformational changes must occur upon binding of a second derCD23 molecule to IgE-Fc.

Despite the undoubted structural asymmetry between the two derCD23 binding sites in IgE-Fc, we cannot definitively assign the stronger and weaker affinity values to these sites. However, it is reasonable to assume that the derCD23 molecule seen in the crystal structure corresponds to the higher affinity site; it also has contributions from Cε2 which could not occur at the site in the other heavy chain. In any event, the binding of the first derCD23 molecule at least preserves, and perhaps even exacerbates, the asymmetry in IgE-Fc and in particular the Cε3-Cε4 interdomain angle; if the latter, then binding to the first site may influence the affinity of the second site (Supplementary Figure S2 and Supplementary Movie). If there is “cross-talk” between the two sites, this is yet another example of communication between receptor binding sites in IgE-Fc, such as that for CD23 and FcεRI. We have previously reported that the binding sites of CD23 and FcεRI on IgE, although at opposite ends of the Cε3 domain, are allosterically linked, whereby conformational changes in IgE upon CD23 binding preclude the binding of FcεRI and *vice versa*^31^. Moreover, Ca^2+^-binding to CD23 causes local allosteric conformational changes that increase its affinity for IgE^20^.

Thus the CD23/IgE/FcεRI network of interactions involves at least two and perhaps three allosteric mechanisms within IgE-Fc. Together these permit an extraordinarily intricate level of control of protein-protein interactions regulating many aspects of the allergic response. Mutual exclusion of FcεRI and CD23 binding to IgE is essential to prevent mast cell and basophil activation by trimeric sCD23 in the absence of allergen^31^. Conformational changes in CD23 on antigen-presenting cells, upon release of bound Ca^2+^, has recently been proposed to be involved in the mechanism that releases IgE/allergen complexes and recycles receptor back to the plasma membrane^43^. On B cells, cross-linking of membrane CD23 by soluble IgE, and/or the formation of extended arrays of mIgE cross-linked by soluble trimeric CD23 to create signaling platforms for the survival and differentiation of IgE-committed cells, have been proposed to control IgE homeostasis^9^. All of these interactions critically depend upon the concentrations and oligomeric states of CD23 and IgE and their mutual affinities. While this study extends our understanding of CD23 binding to IgE-Fc by including the Cε2 domains and their associated conformational changes, it is important to consider the consequences of the presence of the IgE Fab arms, and also possible Fc glycosylation, for the interaction between the complete molecules. We have previously modelled the range of conformations available to the Fab arms in both free and FcεRI-bound IgE, and found that they are more restricted than in IgG, occupying volumes that are mutually exclusive and distinct from the space occupied by the Fc region^44^,^45^. Thus the derCD23/IgE-Fc interaction that we observe in the crystal structure may indeed be extended to the whole IgE molecule. Regarding glycosylation, two sites on the surface of IgE-Fc (asparagine residues 265 and 371) have been shown to be glycosylated^46^, and were mutated to glutamine in the construct used in this study to facilitate crystallization^47^. However, these attachment sites are distant from the CD23 binding site, and therefore unlikely to affect the interaction.

Extrapolation of the results presented here to the binding of either one or two complete CD23 trimers to IgE is also important. We have previously reported that the two head domains that engage Fcε3-4 cannot come from the same trimer^31^, and this is equally true for IgE-Fc or the whole IgE molecule. However, taking all of the above structural considerations into account, it is also clear that two CD23 trimers can bind to one IgE molecule, or one CD23 trimer can cross-link two IgE molecules, either in the context of soluble trimeric CD23 binding to membrane IgE, or soluble IgE binding to membrane CD23, in relation to IgE regulation or allergen/IgE immune complex transcytosis. A detailed molecular understanding of the IgE/CD23 interaction may thus permit novel approaches to intervene therapeutically in allergic disease.

## Methods

### Protein purification

Recombinant human derCD23 (Ser156-Glu298) and IgE-Fc (Val224-Lys547, incorporating glycosylation double mutations N265Q and N371Q) proteins were expressed, refolded and purified according to protocols previously described^15^,^31^,^32^,^47^.

### Crystallization and data collection

The derCD23-IgE-Fc complex was crystallized by vapor diffusion. IgE-Fc was concentrated to 37 mg/mL, and derCD23 to 18 mg/mL, both in 25 mM Tris**·**HCl (pH 7.5), 20 mM NaCl, 0.05% (w/v) sodium azide, followed by mixing of 0.56 mM derCD23 (8.6 mg/mL) with 0.28 mM IgE-Fc (20 mg/mL), and diluting the complex with an equal volume (100 nL) of 15% (w/v) PEG 8,000, 0.1 M sodium citrate, 0.05 M ammonium sulfate. A single needle-like crystal suitable for data collection grew after one and a half months at 295 K. The crystal was flash-cooled (using 18% (w/v) PEG 8,000, 0.1 M sodium citrate, 0.05 M ammonium sulfate, 20% (v/v) glycerol as cryoprotectant) and stored in liquid nitrogen. Diffraction data were collected at 100 K at beamline I03, Diamond Light Source.

### Structure solution and refinement

Indexing and integration of data were carried out with *MOSFLM*^48^,^49^, and merging of data performed using *AIMLESS*^50^. The derCD23-IgE-Fc complex was solved by molecular replacement using *PHASER*^51^; two copies of the Fcε3-4 dimer were identified in the asymmetric unit, using chains A and B from the derCD23-Fcε3-4 complex (PDB 4EZM)^31^. Subsequently, two sets of Cε2 dimers (chains A and B, residues 235–325, PDB 2WQR)^11^ were located. Finally, two Ca^2+^-free derCD23 molecules (using PDB 4J6J)^35^ were identified. Iterative cycles of refinement using *PHENIX*^52^, *REFMAC5*^53^ and *BUSTER-TNT*^54^ alternated with manual model building with *COOT*^55^. The model was built into 2*F*_o_**–***F*_c_ composite omit, 2*F*_o_**–***F*_c_ and *F*_o_**–***F*_c_ electron density maps to minimize bias. Disruption of some disulfide bonds due to radiation damage was observed in the derCD23/IgE-Fc complex structure. Carbohydrate atoms were subsequently incorporated into the structures. During refinement, tight NCS restraints were initially used; these were gradually relaxed and finally local structure similarity restraints^54^ applied. TLS groups^56^ were identified using the *TLSMD* Web server^57^. Data processing and refinement statistics are provided in Supplementary Table S1. *PISA*^58^ and *CONTACTS*^38^ were used to analyze protein-protein interfaces, and *DynDom*^59^ was used for conformational change analysis. All of the structural figures presented were generated using *PyMOL*^60^.

### Isothermal titration calorimetry

The derCD23 and IgE-Fc proteins were dialyzed overnight into 25 mM Tris**·**HCl (pH 7.4), 125 mM NaCl supplemented with 4 mM CaCl_2_^20^. The experiment was carried out at 25 °C in an iTC200 microcalorimeter (Microcal, GE Healthcare). To measure the affinity of derCD23 to IgE-Fc, 260 μM derCD23 was loaded in the syringe and titrated into 12.5 μM of IgE-Fc dimer within the sample cell. Data analysis was carried out using the Origin software supplied with the machine using a 2:1 binding model. Values in parentheses in Table 1 indicate the standard deviation.

## Additional Information

**How to cite this article**: Dhaliwal, B. *et al*. IgE binds asymmetrically to its B cell receptor CD23. *Sci. Rep.* **7**, 45533; doi: 10.1038/srep45533 (2017).

**Publisher's note:** Springer Nature remains neutral with regard to jurisdictional claims in published maps and institutional affiliations.

## Supplementary Material

Supplementary Information

Supplementary Movie M1

## Figures and Tables

**Table 1:** Binding affinities of derCD23 to IgE-Fc studied by ITC.

Experiment	*K*_D1_ (μM)	ΔH_1_ (kcal/mol)	−TΔS_1_ (kcal/mol)	*K*_D2_ (μM)	ΔH_2_ (kcal/mol)	−TΔS_2_ (kcal/mol)
derCD23 titrated into IgE-Fc	1.2 ( ± 0.3)	−7.2 ( ± 0.4)	−0.9	14.4 ( ± 3.0)	−3.7 ( ± 0.6)	−3.0

## References

[b1] Pawankar R., Walter Canonica G., Holgate S. T. & Lockey R. F. World Allergy Organization. White Book on Allergy 2011–2012 Executive Summary (2011).

[b2] DunnGalvin A., Dubois A. E., Flokstra-de Blok B. M. & Hourihane J. O. The effects of food allergy on quality of life. Chem Immunol Allergy 101, 235–252, doi: 10.1159/000375106 (2015). 26022884

[b3] Lifschitz C. The impact of atopic dermatitis on quality of life. Ann Nutr Metab 66 Suppl 1, 34–40, doi: 10.1159/000370226 (2015). 25925339

[b4] Meltzer E. O. Allergic Rhinitis: Burden of Illness, Quality of Life, Comorbidities, and Control. Immunol Allergy Clin North Am 36, 235–248, doi: 10.1016/j.iac.2015.12.002 (2016). 27083099

[b5] Sharma H. P. & Herbert L. J. Food allergy: psychosocial impact and public policy implications. Chem Immunol Allergy 101, 221–226, doi: 10.1159/000373907 (2015). 26022882

[b6] Gupta R. . The economic impact of childhood food allergy in the United States. JAMA Pediatr 167, 1026–1031, doi: 10.1001/jamapediatrics.2013.2376 (2013). 24042236

[b7] Mir E., Panjabi C. & Shah A. Impact of allergic rhinitis in school going children. Asia Pacific allergy 2, 93–100, doi: 10.5415/apallergy.2012.2.2.93 (2012). 22701858 PMC3345332

[b8] Bock S. A., Munoz-Furlong A. & Sampson H. A. Fatalities due to anaphylactic reactions to foods. J Allergy Clin Immunol 107, 191–193, doi: 10.1067/mai.2001.112031 (2001). 11150011

[b9] Gould H. J. & Sutton B. J. IgE in allergy and asthma today. Nature reviews. Immunology 8, 205–217, doi: 10.1038/nri2273 (2008). 18301424

[b10] Sutton B. J. & Davies A. M. Structure and dynamics of IgE-receptor interactions: FcepsilonRI and CD23/FcepsilonRII. Immunol Rev 268, 222–235, doi: 10.1111/imr.12340 (2015). 26497523

[b11] Holdom M. D. . Conformational changes in IgE contribute to its uniquely slow dissociation rate from receptor FcεRI. Nature structural & molecular biology 18, 571–576, doi: 10.1038/nsmb.2044 (2011). PMC3357048 21516097

[b12] Voehringer D. Protective and pathological roles of mast cells and basophils. Nature reviews. Immunology 13, 362–375, doi: 10.1038/nri3427 (2013). 23558889

[b13] Zelensky A. N. & Gready J. E. The C-type lectin-like domain superfamily. The FEBS journal 272, 6179–6217, doi: 10.1111/j.1742-4658.2005.05031.x (2005). 16336259

[b14] Beavil A. J., Edmeades R. L., Gould H. J. & Sutton B. J. Alpha-helical coiled-coil stalks in the low-affinity receptor for IgE (Fc epsilon RII/CD23) and related C-type lectins. Proceedings of the National Academy of Sciences of the United States of America 89, 753–757 (1992). 1309956 10.1073/pnas.89.2.753 PMC48317

[b15] Hibbert R. G. . The structure of human CD23 and its interactions with IgE and CD21. The Journal of experimental medicine 202, 751–760, doi: 10.1084/jem.20050811 (2005). 16172256 PMC2212946

[b16] Chen B. H. . Necessity of the stalk region for immunoglobulin E interaction with CD23. Immunology 107, 373–381 (2002). 12423314 10.1046/j.1365-2567.2002.01512.x PMC1782806

[b17] Dierks S. E. . The oligomeric nature of the murine Fc epsilon RII/CD23. Implications for function. J Immunol 150, 2372–2382 (1993). 8450218

[b18] Kelly A. E., Chen B. H., Woodward E. C. & Conrad D. H. Production of a chimeric form of CD23 that is oligomeric and blocks IgE binding to the Fc epsilonRI. Journal of immunology 161, 6696–6704 (1998). 9862699

[b19] McCloskey N. . Soluble CD23 monomers inhibit and oligomers stimulate IgE synthesis in human B cells. The Journal of biological chemistry 282, 24083–24091, doi: 10.1074/jbc.M703195200 (2007). 17576766

[b20] Yuan D. . Ca^2+^-dependent Structural Changes in the B-cell Receptor CD23 Increase Its Affinity for Human Immunoglobulin E. The Journal of biological chemistry 288, 21667–21677, doi: 10.1074/jbc.M113.480657 (2013). 23775083 PMC3724626

[b21] Weskamp G. . ADAM10 is a principal ‘sheddase’ of the low-affinity immunoglobulin E receptor CD23. Nature immunology 7, 1293–1298, doi: 10.1038/ni1399 (2006). 17072319

[b22] Schulz O. . Cleavage of the low-affinity receptor for human IgE (CD23) by a mite cysteine protease: nature of the cleaved fragment in relation to the structure and function of CD23. Eur J Immunol 27, 584–588, doi: 10.1002/eji.1830270303 (1997). 9079796

[b23] Acharya M. . CD23/FcepsilonRII: molecular multi-tasking. Clin Exp Immunol 162, 12–23, doi: 10.1111/j.1365-2249.2010.04210.x (2010). 20831712 PMC2990925

[b24] Tu Y. . CD23-mediated IgE transport across human intestinal epithelium: inhibition by blocking sites of translation or binding. Gastroenterology 129, 928–940, doi: 10.1053/j.gastro.2005.06.014 (2005). 16143132

[b25] Palaniyandi S., Tomei E., Li Z., Conrad D. H. & Zhu X. CD23-dependent transcytosis of IgE and immune complex across the polarized human respiratory epithelial cells. Journal of immunology 186, 3484–3496, doi: 10.4049/jimmunol.1002146 (2011). 21307287

[b26] Mudde G. C., Reischul I. G., Corvaia N., Hren A. & Poellabauer E. M. Antigen presentation in allergic sensitization. Immunol Cell Biol 74, 167–173, doi: 10.1038/icb.1996.23 (1996). 8724005

[b27] van der Heijden F. L., Joost van Neerven R. J., van Katwijk M., Bos J. D. & Kapsenberg M. L. Serum-IgE-facilitated allergen presentation in atopic disease. Journal of immunology 150, 3643–3650 (1993). 8468493

[b28] Selb R. . CD23 surface density on B cells is associated with IgE levels and determines IgE-facilitated allergen uptake, as well as activation of allergen-specific T cells. J Allergy Clin Immunol, doi: 10.1016/j.jaci.2016.03.042 (2016). PMC5321593 27372566

[b29] Fellmann M., Buschor P., Rothlisberger S., Zellweger F. & Vogel M. High affinity targeting of CD23 inhibits IgE synthesis in human B cells. Immun Inflamm Dis 3, 339–349, doi: 10.1002/iid3.72 (2015). 26732048 PMC4693728

[b30] Yabuuchi S., Nakamura T., Kloetzer W. S. & Reff M. E. Anti-CD23 monoclonal antibody inhibits germline Cepsilon transcription in B cells. Int Immunopharmacol 2, 453–461 (2002). 11962725 10.1016/s1567-5769(01)00187-4

[b31] Dhaliwal B. . Crystal structure of IgE bound to its B-cell receptor CD23 reveals a mechanism of reciprocal allosteric inhibition with high affinity receptor FcepsilonRI. Proceedings of the National Academy of Sciences of the United States of America 109, 12686–12691, doi: 10.1073/pnas.1207278109 (2012). 22802656 PMC3412039

[b32] Shi J. . Interaction of the low-affinity receptor CD23/Fc epsilonRII lectin domain with the Fc epsilon3-4 fragment of human immunoglobulin E. Biochemistry 36, 2112–2122, doi: 10.1021/bi961231e (1997). 9047310

[b33] Wan T. . The crystal structure of IgE Fc reveals an asymmetrically bent conformation. Nature immunology 3, 681–686, doi: 10.1038/ni811 (2002). 12068291

[b34] Borthakur S. . Mapping of the CD23 binding site on IgE and allosteric control of the IgE-FcεRI interaction. The Journal of biological chemistry, doi: 10.1074/jbc.C112.397059 (2012). PMC3438978 22815482

[b35] Dhaliwal B. . Conformational plasticity at the IgE-binding site of the B-cell receptor CD23. Mol Immunol 56, 693–697, doi: 10.1016/j.molimm.2013.07.005 (2013). 23933509 PMC3807792

[b36] Dhaliwal B., Pang M. O., Yuan D., Beavil A. J. & Sutton B. J. A range of Cε3-Cε4 interdomain angles in IgE Fc accommodate binding to its receptor CD23. Acta crystallographica. Section F, Structural biology communications 70, 305–309, doi: 10.1107/S2053230X14003355 (2014). 24598915 PMC3944690

[b37] Lawrence M. C. & Colman P. M. Shape complementarity at protein/protein interfaces. Journal of molecular biology 234, 946–950, doi: 10.1006/jmbi.1993.1648 (1993). 8263940

[b38] Winn M. D. . Overview of the CCP4 suite and current developments. Acta crystallographica. Section D, Biological crystallography 67, 235–242, doi: 10.1107/S0907444910045749 (2011). 21460441 PMC3069738

[b39] Beavil A. J., Young R. J., Sutton B. J. & Perkins S. J. Bent domain structure of recombinant human IgE-Fc in solution by X-ray and neutron scattering in conjunction with an automated curve fitting procedure. Biochemistry 34, 14449–14461 (1995). 7578050 10.1021/bi00044a023

[b40] Wurzburg B. A., Garman S. C. & Jardetzky T. S. Structure of the human IgE-Fc Cε3-Cε4 reveals conformational flexibility in the antibody effector domains. Immunity 13, 375–385 (2000). 11021535 10.1016/s1074-7613(00)00037-6

[b41] Wurzburg B. A. & Jardetzky T. S. Conformational flexibility in immunoglobulin E-Fc 3-4 revealed in multiple crystal forms. Journal of molecular biology 393, 176–190, doi: 10.1016/j.jmb.2009.08.012 (2009). 19682998 PMC2827403

[b42] Garman S. C., Wurzburg B. A., Tarchevskaya S. S., Kinet J. P. & Jardetzky T. S. Structure of the Fc fragment of human IgE bound to its high-affinity receptor Fc epsilonRI alpha. Nature 406, 259–266, doi: 10.1038/35018500 (2000). 10917520

[b43] Andersen C. B. & Moestrup S. K. How calcium makes endocytic receptors attractive. Trends in biochemical sciences 39, 82–90, doi: 10.1016/j.tibs.2013.12.003 (2014). 24393667

[b44] Hunt J. . A fluorescent biosensor reveals conformational changes in human immunoglobulin E Fc: implications for mechanisms of receptor binding, inhibition and allergen recognition. The Journal of biological chemistry, doi: 10.1074/jbc.M111.331967 (2012). PMC3366799 22442150

[b45] Drinkwater N. . Human immunoglobulin E flexes between acutely bent and extended conformations. Nature structural & molecular biology 21, 397–404, doi: 10.1038/nsmb.2795 (2014). PMC3977038 24632569

[b46] Plomp R. . Site-specific N-glycosylation analysis of human immunoglobulin E. J Proteome Res 13, 536–546, doi: 10.1021/pr400714w (2014). 24308486

[b47] Young R. J. . Secretion of recombinant human IgE-Fc by mammalian cells and biological activity of glycosylation site mutants. Protein engineering 8, 193–199 (1995). 7543206 10.1093/protein/8.2.193

[b48] Powell H. R. The Rossmann Fourier autoindexing algorithm in MOSFLM. Acta crystallographica. Section D, Biological crystallography 55, 1690–1695 (1999). 10531518 10.1107/s0907444999009506

[b49] Battye T. G., Kontogiannis L., Johnson O., Powell H. R. & Leslie A. G. iMOSFLM: a new graphical interface for diffraction-image processing with MOSFLM. Acta crystallographica. Section D, Biological crystallography 67, 271–281, doi: 10.1107/S0907444910048675 (2011). 21460445 PMC3069742

[b50] Evans P. R. & Murshudov G. N. How good are my data and what is the resolution? Acta crystallographica. Section D, Biological crystallography 69, 1204–1214, doi: 10.1107/S0907444913000061 (2013). 23793146 PMC3689523

[b51] McCoy A. J. . Phaser crystallographic software. J. Appl. Crystallogr. 40, 658–674, doi: 10.1107/S0021889807021206 (2007). 19461840 PMC2483472

[b52] Adams P. D. . The Phenix software for automated determination of macromolecular structures. Methods 55, 94–106, doi: 10.1016/j.ymeth.2011.07.005 (2011). 21821126 PMC3193589

[b53] Murshudov G. N. . REFMAC5 for the refinement of macromolecular crystal structures. Acta crystallographica. Section D, Biological crystallography 67, 355–367, doi: 10.1107/S0907444911001314 (2011). 21460454 PMC3069751

[b54] Smart O. S. . Exploiting structure similarity in refinement: automated NCS and target-structure restraints in BUSTER. Acta crystallographica. Section D, Biological crystallography 68, 368–380, doi: 10.1107/S0907444911056058 (2012). 22505257 PMC3322596

[b55] Emsley P., Lohkamp B., Scott W. G. & Cowtan K. Features and development of Coot. Acta crystallographica. Section D, Biological crystallography 66, 486–501, doi: 10.1107/S0907444910007493 (2010). 20383002 PMC2852313

[b56] Painter J. & Merritt E. A. Optimal description of a protein structure in terms of multiple groups undergoing TLS motion. Acta crystallographica. Section D, Biological crystallography 62, 439–450, doi: 10.1107/S0907444906005270 (2006). 16552146

[b57] Painter J. & Merritt E. A. A molecular viewer for the analysis of TLS rigid-body motion in macromolecules. Acta crystallographica. Section D, Biological crystallography 61, 465–471, doi: 10.1107/S0907444905001897 (2005). 15809496

[b58] Krissinel E. & Henrick K. Inference of macromolecular assemblies from crystalline state. Journal of molecular biology 372, 774–797, doi: 10.1016/j.jmb.2007.05.022 (2007). 17681537

[b59] Hayward S. & Berendsen H. J. Systematic analysis of domain motions in proteins from conformational change: new results on citrate synthase and T4 lysozyme. Proteins 30, 144–154 (1998). 9489922

[b60] Schrödinger L. The PyMOL Graphics System, Version 1.5.0 (2011).

