# Supplementary material for: IgE binds asymmetrically to its B cell receptor CD23
Source: Sci Rep. 2017 Mar 31;7:45533. doi: 10.1038/srep45533 (PMC5374546; doi:10.1038/srep45533)
Supplement: Supplementary Information [file srep45533-s1.pdf]

Supplementary Information

IgE binds asymmetrically to its B cell receptor CD23

Balvinder Dhaliwal[1*], Marie O. Y. Pang[2], Anthony H. Keeble[2,3], Louisa K. James[2,4], Hannah J. Gould[2], James M. McDonnell[2], Brian J. Sutton[2*] and Andrew J. Beavil[2*]

[1]Division of Infection, Immunity and Respiratory Medicine, School of Biological Sciences, Faculty of Biology, Medicine and Health, Manchester Academic Health Sciences Centre and Manchester Institute of Biotechnology, University of Manchester, 131 Princess Street, Manchester, M1 7DN, U.K.
[2]Randall Division of Cell and Molecular Biophysics, Faculty of Life Sciences & Medicine, King's College London, New Hunt's House, Guy's Campus, London, SE1 1UL, U.K. Medical Research Council and Asthma UK Centre in Allergic Mechanisms of Asthma, London, U.K.

Current addresses: -
[3]Department of Biochemistry, University of Oxford, South Parks Road, Oxford, OX1 3QU, U.K.
[4]Blizard Institute, Barts and The London Medical School, Queen Mary University of London, London E1 2AT, U.K.

[*]Corresponding authors: -
balvinder.dhaliwal@manchester.ac.uk Tel. (+44)(0)161 306 4214
brian.sutton@kcl.ac.uk Tel. (+44)(0)20 7848 6423
andrew.beavil@kcl.ac.uk Tel. (+44)(0)20 7848 8064

Supplementary movie legend

Movie M1. Morphing between the crystal structures of free IgE-Fc and IgE-Fc bound to a single derCD23 molecule. The conformational changes shown may represent those occurring in IgE-Fc upon the binding of a single derCD23 molecule. The IgE-Fc $(C\varepsilon 2)_2$ dimer swings out by 16° and the Cε3 domain to which derCD23 binds 'closes' by 13° relative to chain B of free IgE-Fc.

Supplementary figure legends

Figure S1. Venn diagram showing the salt bridge and hydrogen bond interactions observed at the interface of derCD23 in complex with Fcε3-4 or IgE-Fc. Interactions observed in the derCD23/Fcε3-4 crystal structure crystallised in the space group $P2_12_12_1$ can be found within the red ellipsoid; the derCD23/Fcε3-4 interface interactions from the complex crystallised in *P*1 are within the green ellipsoid; derCD23/IgE-Fc interface interactions are within the yellow ellipsoid; Ca^{2+}-bound derCD23/Fcε3-4 interface interactions are within the blue ellipsoid. The total number of salt bridges and hydrogen bonds observed at each interface are nine, ten, eleven and thirteen, respectively. Notably, one Cε2 domain of IgE-Fc only forms a single hydrogen bond with derCD23 namely, Thr260 – His216 of derCD23.

Figure S2. Comparison of Fcε3-4 heavy chains taken from Fcε3-4 and IgE-Fc crystal structures. The Fcε3-4 and IgE-Fc structures compared are: (1) derCD23/IgE-Fc (chains A and B, colored red and blue, respectively, with the Cε2 domains omitted for clarity); (2) derCD23/IgE-Fc (chains C and D, colored orange and cyan, respectively); (3) $P2_12_12_1$ derCD23/Fcε3-4 (chain D, the most closed, colored yellow, PDB ID 4EZM); (4) $P2_12_12_1$ derCD23/Fcε3-4 (chain C, the most "open" when bound to

derCD23, colored green, PDB ID 4EZM); (5) *P*1 derCD23/Fcε3-4 (violet, 4KI1); (6) free IgE-Fc (pale yellow and pale green, 2WQR); (7) Ca^{2+}-bound derCD23/Fcε3-4 (chains D and E, the range observed, colored brown and lime green, respectively, 4GKO); (8) sFcεRIα/Fcε3-4 (chain B, the most open, light orange, 1F6A); (9) sFcεRIα/IgE-Fc (chain B, the most open, pink, 2Y7Q). (a) Comparison of structures 1 and 2. (b) 1, 3 and 4. (c) 1 and 5. (d) 1 and 6. (e) 1 and 7. (f) 1, 8 and 9. (All chains were superposed on the Cε4 domains).

Figure S3. Composite image of the derCD23 and sFcεRIα complexes with Fcε3-4 or IgE-Fc to show the mutual incompatibility of their binding modes. derCD23/Fcε3-4 (PDB ID 4EZM) and sFcεRIα/IgE-Fc (2Y7Q) complexes are superposed onto the 1:1 derCD23/IgE-Fc structure presented here. The second derCD23 molecule from the derCD23/Fcε3-4 complex is colored translucent green, and sFcεRIα from the sFcεRIα/IgE-Fc complex is colored translucent yellow. Steric clashes that would result from the binding of the second derCD23 molecule or sFcεRIα with the 1:1 derCD23/IgE-Fc complex are indicated (orange surfaces).

Figure S4. ITC isotherms showing binding of derCD23 to IgE-Fc.

Supplementary figures

Figure S1

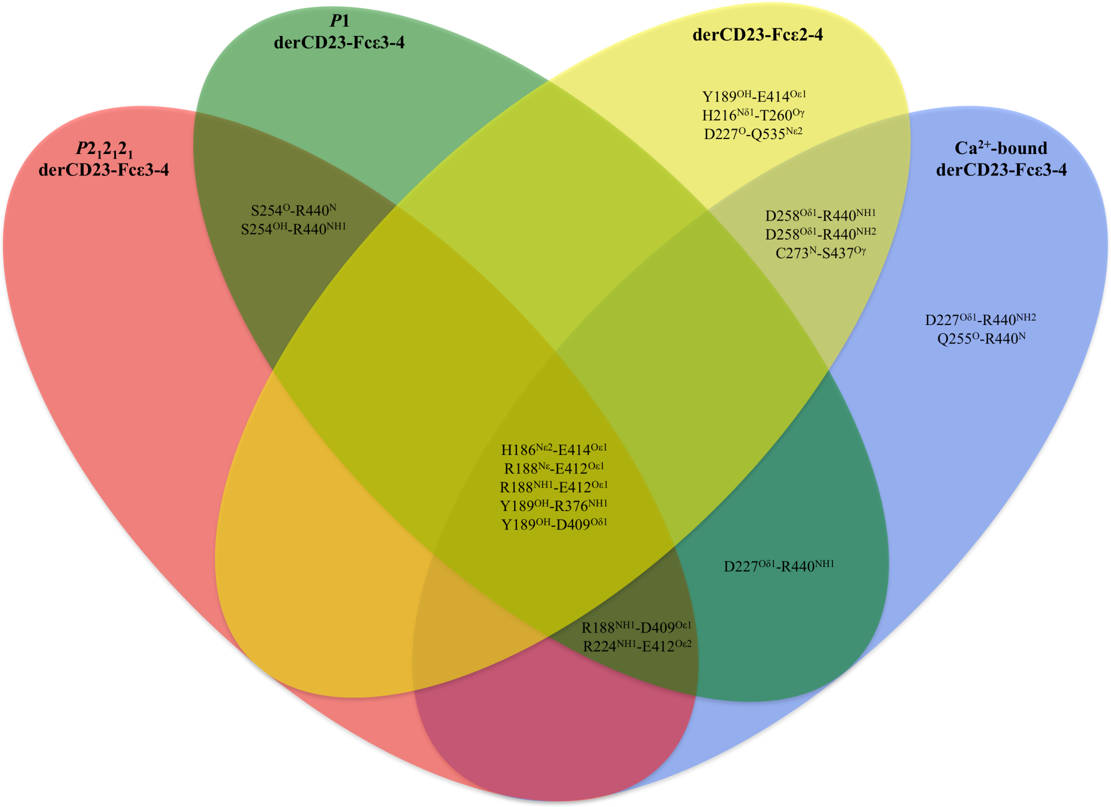

Figure S2

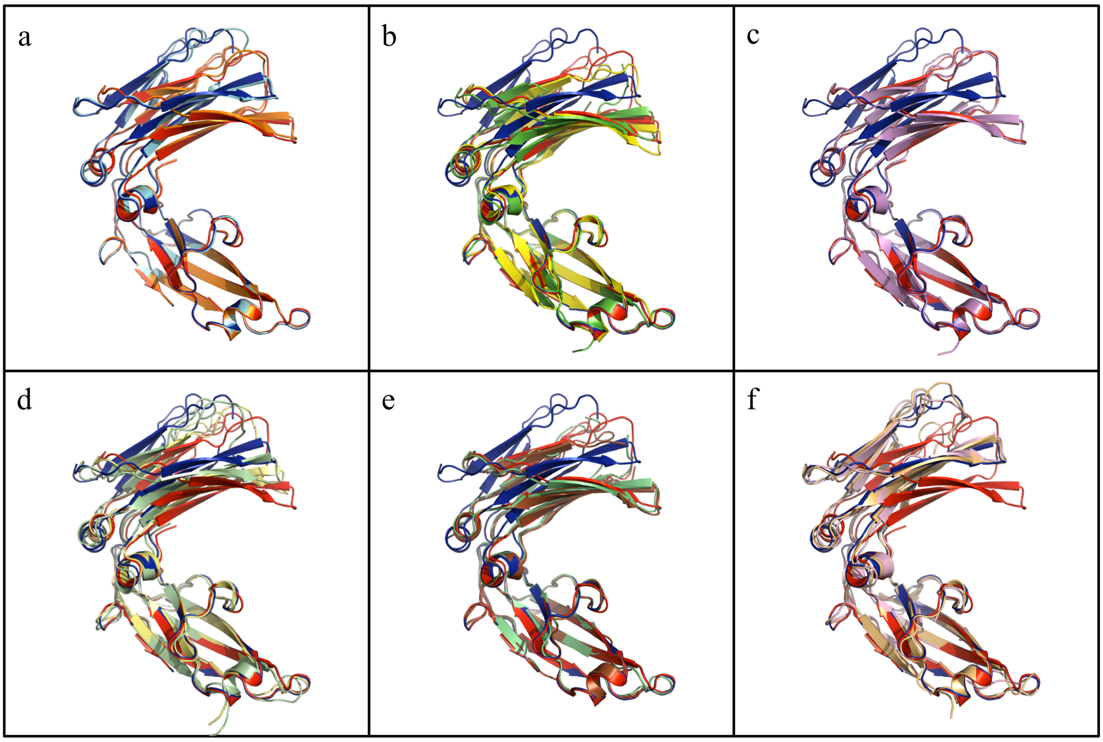

Figure S3

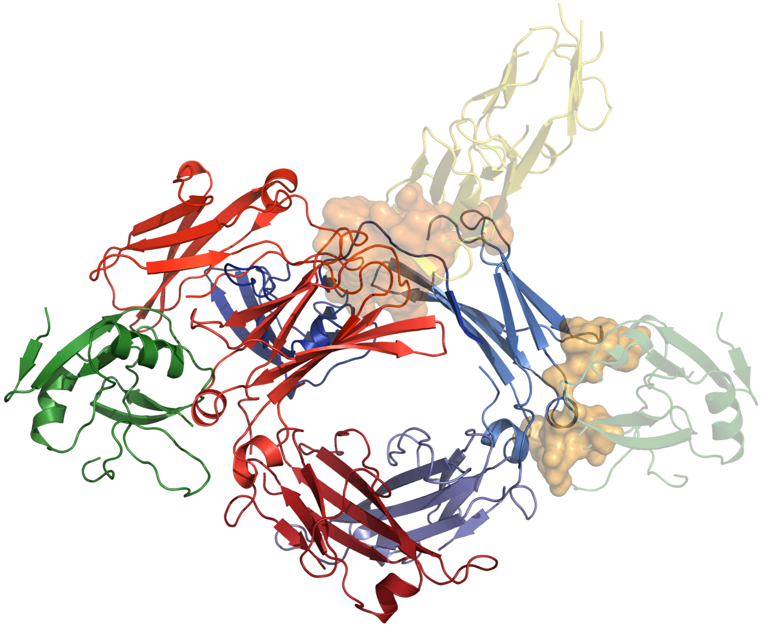

Figure S4

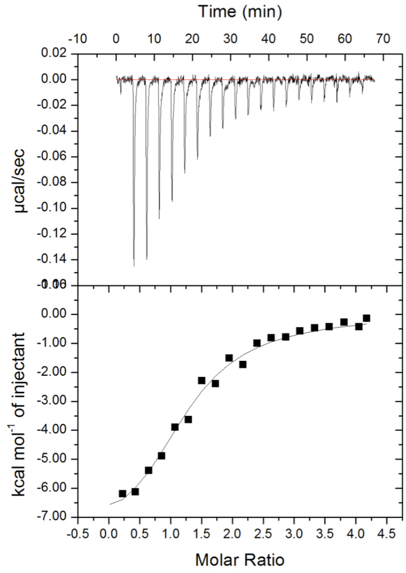
Time (min)
-10 0 10 20 30 40 50 60 70
0.02
0.00
-0.02
-0.04
-0.06
-0.08
-0.10
-0.12
-0.14
µcal/sec
0.00
-1.00
-2.00
-3.00
-4.00
-5.00
-6.00
-7.00
kcal mol-1 of injectant
-0.5 0.0 0.5 1.0 1.5 2.0 2.5 3.0 3.5 4.0 4.5
Molar Ratio

Supplementary Tables

Table S1 Crystallographic data collection and refinement statistics

	derCD23-IgE-Fc
PDB accession code	5LGK
Data processing statistics	
Diamond Light Source beamline	I03
Wavelength (Å)	0.9763
Space group	*P*1
Unit cell parameters (Å)	a = 80.52
	b = 85.32
	c = 88.23
(°)	α = 117.87
	β = 90.32
	γ = 112.62
Number of mols/a.u.	4
Solvent content (%)	52
Resolution range (Å)*	76.0 – 3.50 (3.69 – 3.50)
Observations	35,421
Unique reflections	21,032
Average redundancy	1.7 (1.7)
Completeness (%)	89.3 (88.0)
Wilson B factor (Å^2)	49.1
$I/\sigma(I)$	2.1 (1.2)
$R_{p.i.m.}$†	0.319 (0.758)
Refinement statistics	
Resolution range (Å)	75.9 – 3.50
Total No. of reflections	20,922
No. of working reflections	19,835
No. of test reflections	1,087
R_{xpct}‡	0.266
R_{free}Ψ	0.278
No. of atoms	11,420
Protein	11,314
Carbohydrate	106
R.m.s. bond-length deviation (Å)	0.007
R.m.s. bond-angle deviation (°)	0.84
Mean B factor (Å^2)	36.6
Main chain	35.3
Side Chain	36.9
Carbohydrate	92.9
R.m.s. backbone B factor deviation¶	1.7
Ramachandran statistics (%)‖	
Favored	93.3
Allowed	99.3
Outliers	0.7

*Values in parentheses are for the outer resolution shell.

†$R_{p.i.m.}$ (Precision-indicating merging R factor) = $\Sigma_{hkl}[1/(N-1)]^{1/2}\ \Sigma_i|I_i(hkl) - I(-h\text{-}k\text{-}l)|/\Sigma_{hkl}\Sigma_i I_i(hkl)$[1].

‡$R_{xpct} = \Sigma_{hkl}||F_{obs}| - |F_{xpct}||/\Sigma_{hkl}|F_{obs}|$, where $|F_{obs}|$ and $|F_{xpct}|$ are the observed structure factor amplitude and the expectation of the model structure factor amplitude, respectively [2].

ΨR_{free} equals the R_{xpct} of test set (5% of the data removed prior to refinement).

¶R.m.s. deviation between B factors for bonded main-chain atoms.

‖As defined by MolProbity[3].

References

1 Weiss, M. S. Global indicators of X-ray data quality. *J. Appl. Crystallogr.* **34**, 130-135 (2001).

2 Blanc, E. *et al.* Refinement of severely incomplete structures with maximum likelihood in BUSTER-TNT. *Acta crystallographica. Section D, Biological crystallography* **60**, 2210-2221, doi:10.1107/S0907444904016427 (2004).

3 Chen, V. B. *et al.* MolProbity: all-atom structure validation for macromolecular crystallography. *Acta crystallographica. Section D, Biological crystallography* **66**, 12-21, doi:10.1107/S0907444909042073 (2010).
